# Global open data management in metabolomics

**DOI:** 10.1016/j.cbpa.2016.12.024

**Published:** 2017-02

**Authors:** Kenneth Haug, Reza M Salek, Christoph Steinbeck

**Affiliations:** European Bioinformatics Institute (EMBL-EBI), European Molecular Biology Laboratory, Wellcome Trust Genome Campus, Hinxton, Cambridge CB10 1SD, UK

## Abstract

Chemical Biology employs chemical synthesis, analytical chemistry and other tools to study biological systems. Recent advances in both molecular biology such as next generation sequencing (NGS) have led to unprecedented insights towards the evolution of organisms’ biochemical repertoires. Because of the specific data sharing culture in Genomics, genomes from all kingdoms of life become readily available for further analysis by other researchers. While the genome expresses the potential of an organism to adapt to external influences, the Metabolome presents a molecular phenotype that allows us to asses the external influences under which an organism exists and develops in a dynamic way. Steady advancements in instrumentation towards high-throughput and highresolution methods have led to a revival of analytical chemistry methods for the measurement and analysis of the metabolome of organisms. This steady growth of metabolomics as a field is leading to a similar accumulation of big data across laboratories worldwide as can be observed in all of the other omics areas. This calls for the development of methods and technologies for handling and dealing with such large datasets, for efficiently distributing them and for enabling re-analysis. Here we describe the recently emerging ecosystem of global open-access databases and data exchange efforts between them, as well as the foundations and obstacles that enable or prevent the data sharing and reanalysis of this data.

## Highlights

• The metabolome allows accessing the external influences under which an organism exists and develops in a dynamic way.
• Recent years have seen the establishment of a global network for metabolomics data exchange.
• Global metabolomics data exchange is leading to an exponential growth of publically available metabolomics data for re-analysis.

**Current Opinion in Chemical Biology** 2017, **36**:58–63

This review comes from a themed issue on **Omics**

Edited by **Frank C Schroeder** and **Georg Pohnert**

For a complete overview see the Issue and the Editorial

Available online 13th January 2017

**http://dx.doi.org/10.1016/j.cbpa.2016.12.022**

1367-5931/Published by Elsevier Ltd.

## Introduction

Chemical Biology employs chemical synthesis, analytical chemistry and other tools to study biological systems. Recent advances in both molecular biology such as next generation sequencing (NGS) have led to unprecedented insights towards the evolution of organisms’ biochemical repertoires. Because of the specific data sharing culture in Genomics, genomes from all kingdoms of life become readily available for further analysis by other researchers.

While the genome expresses the potential of an organism to adapt to external influences, the metabolome presents a molecular phenotype that allows us to asses the external influences under which an organism exists and develops in a dynamic way. Those external influences and stimuli are often subsumed under the term Exposome [1]. The metabolome, of course, is complemented in this respect by other molecular phenotypes like those characterised by the products of differential gene expression accessible by RNA sequencing techniques [2].

**Figure 1:**
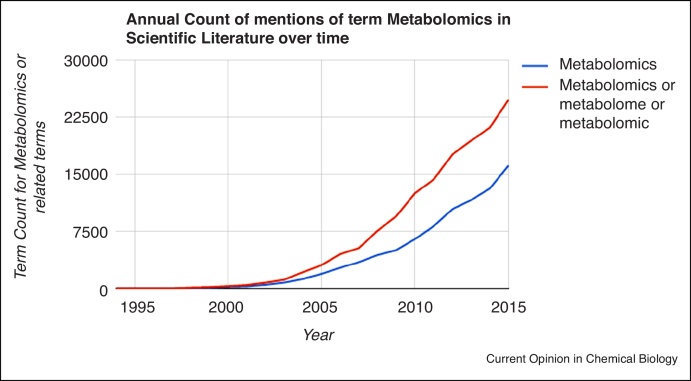
Growth of the occurrence of the term ‘metabolomics’ and synonymous terms in the scientific literature between 1994 and 2015.

Steady advancements in instrumentation towards high-throughput and high-resolution methods have led to a revival of analytical chemistry methods for the measurement and analysis of the metabolome of organisms. Figure 1 demonstrates the steady growth of reported interest in the metabolome through a simple bibliometric analysis on Google Scholar. This steady growth of metabolomics as a field is leading to a similar accumulation of big data across laboratories worldwide as can be observed in all of the other omics areas. This calls for the development of methods and technologies for handling and dealing with such large datasets, for efficiently distributing them and for enabling the re-analysis.

In the following we will describe the recently emerging ecosystem of global open-access databases and data exchange efforts between them, as well as the foundations and obstacles that enable or prevent the data sharing and re-analysis of this data.

## The virtues of data sharing in science

Without progressing into a treatise on the scientific method [3], open data sharing, as well as sharing of open source code and open access to articles, enables scientific peers to reproduce findings reported by a scientist or a group of scientists without barriers. This is important because controlled and/or closed access limits this to specific groups, potentially skewing the efficiency and objectivity of the scientific methods. Learned Societies, funders, some publishers and, in principle, a good portion of the scientific community agree on the importance of data sharing for the advancement of science. This is exemplified by documents such as the Berlin Declaration on Open Access to Knowledge in the Sciences and Humanities [4], which was preceded and followed by many similar texts.

More and more wide-spread acceptance of these principles has led to the creation of a number of organisations and movements to promote the open access to knowledge, information and data, such as the Open Knowledge Foundation [5], the Research Data Alliance (RDA) [6], the Global Alliance for Genomics and Health [7] and more. The virtues of data sharing are at the heart of the scientific method. A scientific publication is indeed not scholarship in itself, but merely an ‘advertisement of scholarship’ [8], whereas the full collection of scientific protocols, materials (this is difficult of course) and underlying data allows peers to assess the validity of the scholarly finding and underlying methods. Furthermore, a large collection of research data on a particular technique or subject lends itself to Meta-analysis, which consists of a set of statistical techniques to combine results from several studies. This may reveals insights that could not have been deduced from a single or only a few datasets but of course also poses questions about reproducibility and comparability caused by different experimental designs [9].

For most of the history of modern science, the sharing of data was done on request by researcher Alice to another scientist Bob who produced the data—with all its social implications. Such requests could be ignored and or access selectively granted, based on Alice’s standing with Bob. The emergence of the internet has the power to remove those barriers, but imposes new challenges. The development of tools and resources for publishing open data is increasingly important and relevant. Increasingly large, heterogeneous, and complex datasets require extra effort for storing, exchanging, and making sense of data. Initiatives to develop these tools and standards are driven by a range of international collaborations, government initiatives, institutions, and local communities. In the major omics areas like genomics, proteomics, and metabolomics, primary research data is being collected in centralised repositories maintained by specialised institutions such as the European Bioinformatics Institute (EMBL-EBI) [10] and the National Center for Biotechnology Information (NCBI) [11]. These institutions have been equipped with a mandate to support data repositories over longer periods of time, outside the usual 3- or 5-year funding cycles. Genomics researchers established guidelines for the deposition of sequence data in 1996 with the creation of the Bermuda Principles [12]. In metabolomics, we have now laid the foundations following on the steps of these pioneering efforts. Global and long-term supported databases exist as well as minimum information standards and procedures for data dissemination [45].

## Global data management in metabolomics

Very few application and domain-specific databases to capture and disseminate primary data in metabolomics have arisen in 90s [13, 14], followed by the establishment of a first round of standardisation efforts by the Metabolomics Standards Initiative (MSI) [15]. Those are complemented by reference databases with information on chemical structures, physicochemical properties, biological functions, pathway network, and most importantly, reference spectral data. They can be classified into pathway-centric and compound-centric databases [16]. Examples for a pathway-centric most commonly used in metabolomics are: KEGG [17], Biocyc [18], Reactome [19], Wikipathways [20]. Examples for compound-centric databases are BMRB [21], ChEBI [22], ChemSpider [23], GMD [13], HMDB [24], MassBank [25], METLIN [26], NIST [27], and PubChem [28]. Compound-centric resources may contain spectral data. In metabolomics, references compounds are often used for metabolite identification by matching NMR resonance or mass spectral features to those of an unknown compound.

## Databases

**Figure 2:**
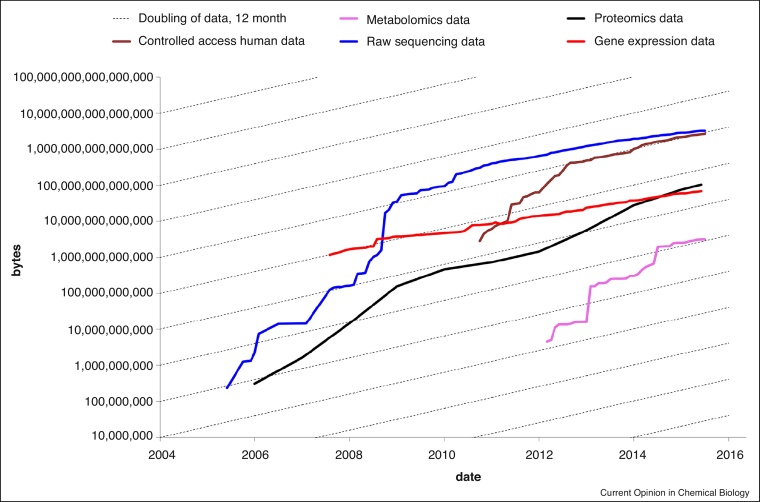
Growth in data repositories at the European Bioinformatics Institute (EMBL-EBI). The graph shows the data volume in each of the repositories over time on a logarithmic scale. Shown are repositories for controlled access human data, raw sequencing data, microarray, proteomics and metabolomics data. Archives were started at different point in history. Metabolomics shows the steepest growth of all repositories at EMBL-EBI.

In the 1990s, global efforts to exchange genomic information [29, 30] arose which eventually evolved into the most liberal model of freely sharing and exchanging data. This led to an unprecedented wave of bioinformatics and biomedical research enabled through the open availability of a growing number of genomes across all kingdoms of life, which still continues to flourish today. It also paved the way for similar efforts in proteomics [31] and gene expression data [32]. In 2012, the European Bioinformatics Institute (EMBL-EBI) launched the MetaboLights database, the first general purpose, cross-species, cross-application database in metabolomics with the aim to enable a similar blossom in this remaining large pillar of omics sciences [33]. In the first two years after its inception, MetaboLights became the fastest growing data repository at the EMBL-EBI in term of data volume (see Figure 2). When the NIH recognised the importance of metabolomics for biomedical research by funding a set of Regional Comprehensive Metabolomics Resource Cores (RCMRC) across the USA, they also decided to invest in a US-based sister repository for MetaboLights, the Metabolomics Workbench [34]. This follows a well-established and −accepted model from genomics and other biomolecular data types of establishing sister repositories in major geographic regions of the world. Those repositories typically collaborate on the data maintenance and data exchange but compete in the way the data presented to their users.

### MetaboLights

The MetaboLights database and repository was the first cross-species, general purpose repository for metabolomics data. Launched in 2012 by the European Bioinformatics Institute (EMBL-EBI) [35], it has seen steady growth in number of submissions, with each submission currently averaging about 20 GB per study, accumulating to about 4 TB of data in May 2016. It covers metabolite structures and their reference spectra, as well as the biological roles, locations, concentrations and experimental data from metabolic experiments. MetaboLights includes user submission tools, and incorporates de-facto standard formats for encoded spectral and chromatographic data, associated information about chemical structures, and metadata for describing assays and studies as a whole. Studies submitted to MetaboLights are manually curated and improved, if necessary, in collaboration with the submitters [36].

Many funders now require data arising from publicly funded organizations to be made freely accessible. The experimental data that scientists submit to MetaboLights have been used to justify findings in scientific studies and to verify experimental methods in peer-reviewed publications. Journal recommend or require the deposition of data in MetaboLights or its sister databases. They therefore play an important role in enabling the transparent reproduction and re-use of metabolomics results. MetaboLights is now the fastest growing repository at the EMBL-EBI, with a 3-month doubling time (see Figure 2)

**Figure 3:**
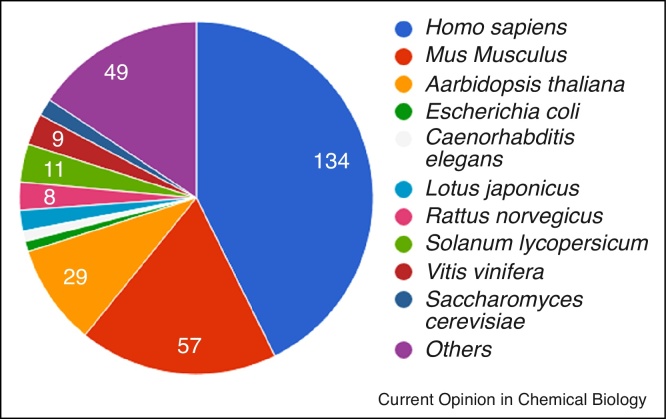
Number of studies in MetaboLights by species. The distribution is reflecting the most used model species in biological and biomedical research.

Figure 3 shows the coverage of species and experimental techniques in MetaboLights. For the core model species in metabolomics, the amount of data is becoming sufficiently close for meta-analyses, but no such studies have been published to far.

### The metabolomics workbench

The Metabolomics Workbench serves as a national and international repository for metabolomics data and metadata, and also includes data analysis tools and access to metabolite standards, protocols, tutorials, and training. The database was funded by the National Institutes of Health (NIH) Metabolomics Common Fund, with the aim to increase US national capacity in metabolomics: by supporting the development of next generation technologies, providing training, enhancing the availability of high quality reference standards, and promoting data sharing and collaboration [34].

The Metabolomics Workbench acts as a North American hub for the metabolomics related research carried out at each of the six Regional Comprehensive Metabolomics Research Cores (RCMRC). All metabolomics research carried out at these centers and funded by the NIH Metabolomics Common Fund must be made publically available via the Metabolomics Workbench. The emerging network of global and long-term supported metabolomics data repositories triggered the need for a global service to discover the metabolomics data sets regardless of which database they are actually located in.

### MetabolomeXchange

MetabolomeXchange aggregates data from three different data providers—MetaboLights, Metabolomics Workbench and Metabolomic Repository Bordeaux—which together make up the MetabolomeXchange Consortium http://www.metabolomexchange.org/. The goal of MetabolomeXchange is to increase the accessibility of and awareness about newly released, publicly available metabolomics datasets from verified members of the Consortium. MetabolomeXchange aims to provide a network of stable and coordinated metabolomics data, while also assuring that both the scientific community and the commercial user community have access to high-quality reference data. The data “exchanged” through MetabolomeXchange consists of both experimental data and metadata for individual metabolites and metabolomic profiles.

MetabolomeXchange enables researchers to submit data either by submitting to the existing data repositories within the MetabolomeXchange Consortium, or by becoming a data provider and member of the consortium. MetabolomeXchange was launched in 2014, and is coordinated by the EMBL-EBI. It is an outcome of the European-Commission-funded Coordination of Standards in Metabolomics (COSMOS) project [38], which ran from 2012 to 2015, and gathered European metabolomics data providers to establish and promote community standards for metabolomics data and experiments [37]. MetabolomeXchange is modelled on the ProteomeXchange [31], a consortium established in 2012 to provide a coordinated submission of mass-spectrometry proteomics data to the main existing proteomics repositories, and to encourage optimal data dissemination. At the time of writing (December 2016) more than 540 datasets where publicly available on MetabolomeXchange.org.

## Data sharing needs standards

In order to enable both the re-use of data as well as its barrier-free exchange, data and meta-data stored in public repositories such as Metabolomics Workbench or MetaboLights need to be encoded using community-agreed standards [37]. A first round of standardisation efforts in Metabolomics was achieved by the Metabolomics Standards Initiative (MSI) [15]. Around the year 2006, the MSI published documents about the Core Information for Metabolomics Reporting (CIMR). CIMR recommendations were published in the areas of *In Vivio*/Mammalian Biology, Plant Biology, *In Vitro* Biology/Microbiology as well as Environmental Analysis. Those documents are accessible via http://www.metabolomics-MSI.org.

When MetaboLights appeared in 2012, and later Metabolomics Workbench, the field had advanced by six years with new instrumentation and changing protocols. New open data standards had emerged and others were missing. This led to the foundation of the COSMOS initiative for the Coordination of Standards in Metabolomics [38]. Apart from reviving the interest in data and meta-data standards in metabolomics and providing a platform for discussions, COSMOS set out to develop missing open data formats and promote the use of data formats such as mzML [39] and mzTab [40], which had been developed by the proteomics community and could be applied to metabolomics with moderate effort. The recommendations of the MSI on which data to report is nowadays backed by a rich set of ontologies and controlled vocabularies which help researcher speak a common language and to avoid naming diversity through different conventions in different laboratories or communities [41].

To structure data captured according to MI standards and backed by ontologies, the ISA-TAB format [42] and related ecosystem of tools [43] has emerged as a quasi-standards. ISA stands for Investigation-Study-Assay—the typical hierarchical organisation of a biological study. ISA-TAB is a tabular format to hold data in a spreadsheet-like way, in addition offering support for ontologies and much more. Databases like MetaboLights support uploading of study information in ISA-TAB format.

The field of metabolomics continues to evolve new data standards and methods as it progresses. Recently, for example, a hashed identifier for mass spectra, SPLASH, was published, which improves the exchange of mass spectra and allows for the determination of provenance and duplicate detection [44].

## Conclusion

Publishers, funders and learned societies more and more require the open availability of research data and resulting publications. Foundations have been laid to enable the global sharing and long term preservation of research data in metabolomics, following in the footsteps of the other large pillars of biomolecular data science. Deposition of research data in MetaboLights or Metabolomics Workbench will be easier for those laboratories with a structured internal approach to capturing and storing experimental data. In addition to the minimum information standards and data formats to encode primary research data and their meta-data, an ecosystem of tools exists to support the assembly and uploading of the information. Metabolomics data volume in public repositories is growing exponentially and will enable meta- and re-analyses previously not possible.
